# COVID-19 Contact-Tracing Apps: Analysis of the Readability of Privacy Policies

**DOI:** 10.2196/21572

**Published:** 2020-12-03

**Authors:** Melvyn Zhang, Aloysius Chow, Helen Smith

**Affiliations:** 1 Family Medicine and Primary Care Lee Kong Chian School of Medicine Nanyang Technological University Singapore Singapore Singapore

**Keywords:** COVID-19, smartphone apps, contact tracing, privacy policy, readability, app, privacy, surveillance

## Abstract

Apps that enable contact-tracing are instrumental in mitigating the transmission of COVID-19, but there have been concerns among users about the data collected by these apps and their management. Contact tracing is of paramount importance when dealing with a pandemic, as it allows for rapid identification of cases based on the information collected from infected individuals about other individuals they may have had recent contact with. Advances in digital technology have enabled devices such as mobile phones to be used in the contract-tracing process. However, there is a potential risk of users’ personal information and sensitive data being stolen should hackers be in the near vicinity of these devices. Thus, there is a need to develop privacy-preserving apps. Meanwhile, privacy policies that outline the risk associated with the use of contact-tracing apps are needed, in formats that are easily readable and comprehensible by the public. To our knowledge, no previous study has examined the readability of privacy policies of contact-tracings apps. Therefore, we performed a readability analysis to evaluate the comprehensibility of privacy policies of 7 contact-tracing apps currently in use. The contents of the privacy policies of these apps were assessed for readability using Readability Test Tool, a free web-based reliability calculator, which computes scores based on a number of statistics (ie, word count and the number of complex words) and indices (ie, Flesch Reading Ease, Flesch-Kincaid Reading Grade Level, Gunning Fog Index, and Simplified Measure of Gobbledygook index). Our analysis revealed that explanations used in the privacy policies of these apps require a reading grade between 7 and 14, which is considerably higher than the reading ability of the average individual. We believe that improving the readability of privacy policies of apps could be potentially reassuring for users and may help facilitate the increased use of such apps.

Contact tracing is of paramount importance when dealing with a pandemic such as COVID-19. It allows for the rapid identification of cases based on the information collected from infected individuals about their recent contact with other individuals [1]. Additionally, contact-tracing systems allow for the collection of further information about these contacts, in order to help minimize the spread of the disease [2]. Different contact tracing apps rely on different technologies, including GPS, Bluetooth, and millimeter-wave communication technologies. Conventionally, public health care workers can assist an infected patient to map out individuals with whom they might have been in close contact recently, and these individuals are then informed of their susceptibility to the infection. Thus, contact tracing enables the identification of potential cases and allows for the follow-up and rapid quarantining of susceptible individuals [1]. With advances in digital technology, devices such as a mobile phone can now be employed in the contact-tracing process. For instance, a recent article in *Nature* discusses 3 apps developed to rapidly identify contacts of patients with COVID-19, including an Australia-based app called COVIDSafe and similar apps being used in Germany and Egypt [3].

Apps that enable contact tracing are instrumental in response to a public health emergency, but there have been concerns about the data they collect and how they are managed. Although there are potential benefits of using these apps, there are also ongoing concerns. For instance, in a recent commentary, Sharma et al [4] outlined the existing apps for COVID-19 contact tracing and concerns about data privacy. Another article in *Nature* cautioned against the accuracy of such contact-tracing apps and highlighted how these apps might render individuals susceptible to security breaches, given that most of these apps tap on Bluetooth functionality, potentially compromising the exchange of information [5]. This is an inherent risk that personal information and other sensitive personal data might be stolen if hackers happen to be in the vicinity of these devices [5]. A mixed-methods study in Norway analyzed the personal dataflows and the contents of privacy policies of 21 popular, free-to-use Android mobile apps [6]. Their results showed that 19 of the 21 apps studied transmitted personal data to about 600 different primary and third-party domains that were associated with tech companies in the United States. They also found that some apps tracked and shared data by default even when the app was not in use. The terms of use of some of these apps did not inform the users about the data sharing.

This study highlights critical ethical issues of data protection, security, and privacy of data collated by smartphone apps [1] and the consequent need to develop privacy-preserving smartphone apps [7]. A scoping review of the privacy assessments of mobile health apps reported that the evaluation criteria used in studies have been heterogeneous and lacked objectivity [8]. This further emphasizes the need for a common evaluation tool to ensure that personal health data privacy is safeguarded. It has also been suggested that a “healthcare fiduciary” be developed to enhance international regulatory frameworks to increase data protection security [9].

While we await the development of such privacy-preserving apps, privacy policies outlining the risks associated with the use of contact-tracing apps are needed, in a format that can be easily read and comprehended by the public. Readability of policy terms can be evaluated using validated tools that assess the complexity of the vocabulary and syntax, as well as the presentation of the content [10]. In other areas of health care, researchers have started to critique the readability of privacy policies. For instance, Robillard et al [10] focused on the availability and readability of privacy-related content of mental health apps and reported that most apps they studied did not include terms of agreement or a privacy policy. On the other hand, among the apps that had such policies in place, a reading ability more advanced than secondary education was required to comprehend the information. In relation to COVID-19, Basch et al [11] examined the information available on the internet and found that the readability levels required to comprehend the information exceeded that of the average American. The fact that higher-than-average readability levels are required to comprehend web-based information implies that the available information cannot be disambiguated, which might result in increased panic among the app users [11].

Given this situation, we performed a readability analysis of the privacy policies of 7 contact-tracing apps, namely COVIDSafe (used in Australia) [12], BeAware (used in Bahrain) [13], CoronApp (used in Colombia) [14], GH COVID-19 Tracker (used in Ghana) [15], Rakning C-19 (used in Iceland) [16], NZ COVID Tracer (used in New Zealand) [17], and TraceTogether (used in Singapore) [18]. As previously highlighted by Basch et al [11], the provision of timely information, in a format that could be comprehended easily, would help individuals understand important information relevant to the pandemic and, in turn, allay any anxieties. A readability analysis of privacy policies is timely and pertinent, given the considerable number of contact-tracing apps now available and government agencies’ enforcement that individuals download and use these apps. As a result, individuals are now more likely to examine the privacy policies of the apps they use, to understand what data is being shared and how their personal information is being protected. Any difficulty in comprehending the information contained within these privacy policies could result in a reluctance to download and use such apps.

Readability statistics of the privacy policies of the identified apps were computed using Readability Test Tool, a web-based reliability calculator [19]. This free resource computes the word count, Flesch Reading Ease, Flesch-Kincaid Reading Grade Level, Gunning Fog Index, Simplified Measure of Gobbledygook (SMOG) index, and the number of complex words [20]. For this evaluation, we used well-validated methods, based on previous studies that have examined readability [21,22]. The Flesch Reading Ease test evaluates the length of sentences and the number of polysyllabic words to determine the overall readability score; the score ranges from 0 to 100, with a higher score suggesting that the text is easy to read. The Flesh-Kincaid Reading Grade Level test evaluates the mean sentence and word length to compute reading complexity of the text; the score ranges from 1 to 12, corresponding to the US educational school grades, with scores higher than 12 indicative of college-level education and domain-specific experts. The Gunning Fog Index estimates the number of years of formal education required for an individual to understand the text on the first reading; the score ranges from 0 to 19+ and is representative of the readability level of the document. A Gunning Fog score of 0-6 is indicative of low literacy, a score of 7 or 8 is indicative of junior high school–level literacy, a score of 9-12 is indicative of high school–level literacy, a score of 13-16 is indicative of college-level literacy, a score of 17 or 18 is indicative of graduate-level literacy, and a score ≥19 suggests higher professional–level qualifications [23]. The SMOG index estimates the years of education needed to understand a piece of writing, by evaluating 10 sentences from the beginning, middle, and end of the document. The number of syllables in each section is then totaled and converted to a grade-level score [20]. Table 1 shows the readability scores for each of the 7 apps studied.

**Table 1:** Readability scores for the privacy policies of different COVID-19 contact-tracing apps analyzed in this study.

App name and description^a^	Readability scores					
	Total number of words, n	Complexwords, %	Flesch Kincaid Reading Ease	Flesch Kincaid Grade Level	Gunning Fog Score	SMOG index
COVIDSafe [12] Developed by the Australian Government Department of health Uses Bluetooth technology to record any contact one may have had with other users Close contact information is securely stored on the phone, and it can be uploaded and used with the user’s consent	1727	18.8	53.3	8.1	8.9	7.7
BeAware [13] Helps contain COVID-19 spread by advancing contact-tracing efforts Uses location data shared by users to alert individuals if they were in proximity with an active infected case Tracks the movement of quarantine cases Provides updates on COVID-19 developments and latest recommendations issued by health authorities	2893	19.9	45.1	9.1	10.6	8.1
CoronApp [14] Official app by the Government of Chile to prevent COVID-19 spread Allows self-assessment of symptoms Allows user to receive notifications from the Ministry of Health and report high-risk behaviors	4119	20.7	39.9	12.8	14.5	12
GH COVID-19 Tracker [15] Helps individuals assess & self-report symptoms and check risk of infection for COVID-19 Includes the following features: check risk of infection, users near you, self-quarantine management, updates, and event management	2110	16.6	58.2	8.1	9.5	8.2
Rakning C-19 [16] Official app by the Icelandic Government to help mitigate the COVID-19 pandemic in Iceland Collects location information via GPS from the phone and stores data locally on the device Assists in contact tracing	736	16.4	58.8	7.5	8.7	7.5
NZ COVID Tracer [17] Official contact-tracing mobile app by the New Zealand Ministry of Health Assists in contact tracing through the creation of a private digital diary of places the user has visited Provides alerts if the user has checked into a place at the same time as someone with COVID-19	1990	16.3	50.9	11	13	10.3
TraceTogether [18] Supports Singapore’s efforts to fight the spread of COVID-19 through community-driven contact tracing Notifies individuals if they have been exposed to COVID-19 through close contacts Provides the latest guidance from the Singapore Ministry of Health Uses Bluetooth, with the data being stored securely on the phone	645	16.7	48.8	9	9.3	7.8

^a^Each description of the apps have been summarized based on the original app descriptions listed on the app stores.

Users of contact-tracing apps must be aware that the apps gather a lot of their personal data, some from self-reporting and some via sensors in their smartphone devices. Moreover, our findings suggest that the existing explanations in the privacy policies of these apps require a reading level between 7 and 14, which far exceeds many people’s reading ability. Apps like CoronApp [14] and NZ COVID Tracer [17] required the highest-grade level of comprehension (Figure 1), followed by BeAware [13], TraceTogether [18], GH COVID-19 Tracker [15], COVIDSafe [12], and Rakning C-19 [16], listed in order of decreasing readability ease. For example, in the United States, the average reading level is between grades 7 and 8 [24]. For the information to be accessible and achieve maximum impact among the general population, it should be written at a level no higher than grade 6 [22]. Hence, currently, the privacy policies of all the 7 apps analyzed in this study are considered “very difficult” to read and comprehend for the majority of individuals. In their analysis of the readability of online websites on COVID-19, Basch at al [11] highlighted how heightened levels of anxiety about the pandemic might further impair the understanding and interpretation of information, thus exacerbating fear.

**Figure 1:**
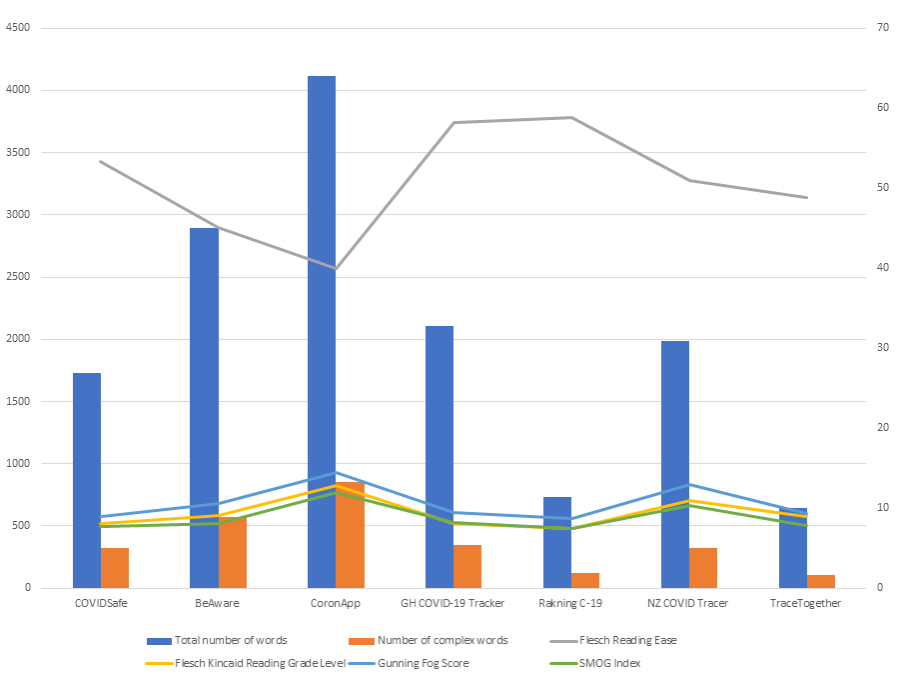
Overview of readability scores for each COVID-19 contact-tracing app evaluated.

With more countries now exiting lockdowns, the use of contact-tracing apps will become more commonplace. While we await improvements to existing apps through the use of more secured technologies, the public must have access to readable terms of agreement or privacy policies to be aware of how their data are being collected, stored, and used. Improving the readability of privacy policies could be reassuring and could facilitate the adoption and eventual impact of these apps. Our study has highlighted COVID-19 contact-tracing apps with privacy policies that are readily understandable by the general public. Government agencies need to recognize this and to adapt their privacy policies accordingly, to ensure that every user can readily understand how their data are being stored and shared by the app. At a macro level, health care ministries and organizations could consider enhancing current regulatory frameworks to increase data protection security [9]. This may cause a trickle-down effect to app developers and companies and to the users, for safeguarding personal data.

Several research implications arise from our study findings. We concur with the suggestions by Bahadori et al [23] that researchers could undertake a number of measures to improve app readability. Users are also occasionally involved in the conceptualization of the app and in user testing. With the increase in participatory research, potential users could perhaps be involved in the cocreation and drafting of the privacy policies for such apps. Academics and developers are encouraged to consider the average reading level of the population when they are drafting these policies. As highlighted by Bahadori et al [23], an effective way to do so is to reduce the length of the sentence and target towards a reading level of grade 6. For continued monitoring of user experiences, they also recommend determining whether readability needs to be improved on an ongoing basis. As these areas develop, an objective evaluation tool should also be developed to assess whether sufficient measures have been taken to safeguard the data of mobile app users. By increasing the level of trust that users have in how an app uses their data, more users will be confident of using these apps. This will bode well as health care research drives into the age of big data to improve health care services for everyone.
